# A comprehensive analysis of alcohol and other drug educational resources available in New South Wales, Australia for content, suitability and readability

**DOI:** 10.1186/s13722-025-00615-5

**Published:** 2025-12-02

**Authors:** Aleena Varghese, Rebecca Amanda, Julie Ayre, Kaniz Fatema, Andrew Miles, Gilbert Whitton, Mythily Subramaniam, Amit Arora

**Affiliations:** 1 Discipline of Public Health, School of Medicine, Faculty of Health, Western Sydney University, Campbelltown Campus, Locked Bag 1797, Penrith, NSW 2751 Australia; 2 Health Equity Across Lifespan (HEAL) Research Laboratory, Campbelltown, NSW 2560 Australia; 3 Sydney Health Literacy Lab, School of Public Health, Faculty of Medicine and Health, The University of Sydney, Sydney, Australia; 4 Drug Health Services, South Western Sydney Local Health District, Cabramatta, NSW Australia; 5 Saw Swee Hock School of Public Health, Institute of Mental Health, National University of Singapore, Singapore, Singapore; 6 Translational Health Research Institute, Western Sydney University, Campbelltown Campus, Locked Bag 1797, Penrith, NSW 2751 Australia; 7 Discipline of Child and Adolescent Health, Sydney Medical School, Faculty of Medicine and Health, The University of Sydney, Westmead, NSW 2145 Australia; 8 Oral Health Services, Sydney Local Health District and Sydney Dental Hospital, NSW Health, Surry Hills, NSW 2010 Australia

**Keywords:** Patient education, Alcohol and other drugs, Readability, Health literacy, Content analysis

## Abstract

**Background:**

The utilisation of online evidence-based written educational resources is crucial in addressing problematic alcohol and other drugs (AOD) use through prevention, treatment, and intervention strategies. However, low health literacy among one in five Australian adults raises concerns regarding the effective understanding of health information. This study aims to evaluate the content, suitability, and readability of AOD resources in New South Wales (Australia), recognising the importance of accessible and informative resources in supporting AOD demand reduction strategies.

**Methods:**

In this research, a comprehensive desktop search was conducted to analyse one to two-page AOD resources readily accessible through the internet in New South Wales, published by government and not-for-profit organisations. The content was thoroughly analysed for its coverage of key AOD topics. The Suitability Assessment of Materials (SAM) instrument evaluated visual and written elements, examining aspects like layout, typography, and illustrations. Readability was assessed using Flesch -Kincaid Grade Level (FKGL), Gunning Fog Index (FOG), Simplified Measure of Gobbledygook (SMOG), and Flesch Reading Ease tools. Descriptive statistics, including frequency, percentage, and standard deviation were calculated.

**Results:**

The study analysed 88 AOD resources. Most resources had a target audience, but only three resources involved consumers in the development process. The content analysis showed 66% focused on drug-related topics, 20% on alcohol-related topics, and 14% covered both. Topics such as alcohol use during pregnancy and breastfeeding were well addressed in alcohol resources. Additionally, 90% of the resources had headings and subheadings. However, only 28% scored ‘superior’ for layout, and none achieved ‘superior’ ratings for typography. Furthermore, 74% did not use illustrations to highlight key messages. Most resources used an active voice and conversational style, but complex sentences were common. The average reading grade level of the resources was 9 *±* 2.6 with FOG and Flesch’s reading ease indicating 10th-grade difficulty, while FKGL and SMOG suggested a 7th-grade level.

**Conclusions:**

The evidence strongly suggests the need for the development of AOD resources that are accessible to individuals with low literacy levels without sacrificing content coverage. A key recommendation is to involve consumers in both developing and reviewing these resources.

**Supplementary Information:**

The online version contains supplementary material available at 10.1186/s13722-025-00615-5.

## Background

The use of alcohol and other drugs (AOD) is a significant contributor to avoidable health issues, morbidity, and mortality in the world [1]. According to United Nations reports, approximately 35 million individuals suffer from substance abuse disorders globally [2]. Each year, alcohol consumption causes 3 million deaths on a global scale and contributes to 5.1% of the global burden of disease [3].

In Australia, the prevalence of AOD use is substantial. Alcohol was consumed by 77% of Australians aged 14 and older from 2022 to 2023, while 43% of Australians have engaged in the use of illicit drugs during their lifetime [1]. Alcohol consumption is a causal factor in over 200 diseases, including liver cirrhosis, malignancies, communicable diseases such as tuberculosis, pneumonia and HIV/AIDS, as well as cardiovascular diseases [3, 4]. It also contributes significantly to unintentional and intentional injuries such as road accidents, violence, and suicide, particularly prevalent in younger age groups [3]. In 2021, Australia recorded 1,704 drug-induced deaths with approximately 96.7% attributed to acute effects such as overdose, while 3.3% were linked to chronic drug effects, including drug-induced cardiac conditions [1]. These reports suggest that the consequences of consumption of AOD substances encompass a wide spectrum of adverse health effects, socioeconomic burden, and public health issues that extend across different populations and areas, thus necessitating the need for extensive interdisciplinary study and intervention techniques.

In New South Wales (NSW), a collaborative network comprising NSW health services, non-profit organisations and academic research institutes, endeavours to provide a diverse array of services to address the challenges faced by individuals experiencing AOD issues [5]. These services offer a comprehensive treatment framework encompassing withdrawal management, counselling, education,emergency services, rehabilitation and pharmacotherapy-based interventions [5]. An important role of these services is the provision of AOD information and education, disseminated through written materials such as pamphlets, leaflets, and brochures [6]. People struggling with addiction and substance use disorders, or their families and carers, often turn to these online resources for guidance and support [7]. However, for these resources to be effective, they need to be written in a comprehensible manner for the target audience. This necessity underscores the pivotal role of health literacy in comprehending such information, serving as a fundamental element in understanding of health-related content [8].

Health literacy refers to the capacity of individuals to access, comprehend, and effectively utilise health information in a manner that promotes and enhances their overall health and well-being [9, 10]. Studies have found that people with low health literacy have worse health outcomes and increased hospitalisations [11]. Initially centred on individuals’ ability to read and understand medical information, health literacy has expanded to recognise the significance of how information is delivered [12]. For individuals with AOD use disorders, improving health literacy can contribute to reducing disparities in healthcare access and outcomes. By enhancing their ability to comprehend treatment options, navigate healthcare systems, and make informed decisions, health literacy empowers individuals to overcome barriers to quality care and achieve better health outcomes [13]. The Australian Bureau of Statistics (ABS) survey on health literacy revealed that approximately one in six Australians cannot critically evaluate health-related information [14]. A suggested strategy to enhance health literacy involves ensuring that materials are written in a manner comprehensible to the public. This can only be accomplished by meticulously accounting for elements such as the client’s literacy levels, the content’s readability levels, the use of appropriate pictures, and the overall design [6]. Methods employed for readability assessment serve the purpose of anticipating the requisite educational level that an individual must possess to comprehend printed materials and facilitate revision when deemed necessary. To address this critical issue, The NSW Health Literacy Framework recommends 6–8 grade readability levels for all print, audio-visual and online health-promotion materials [15]. However, readability testing focuses on analysing the linguistic and structural aspects of written text, neglecting the content, conceptual difficulty, and appropriateness for a specific target audience. Therefore, in the context of written online resources, the content and pictorial presentation of the information also play a crucial role in improving the health literacy of the resources [16–20].

To date, there is a scarcity of research evaluating written educational materials tailored for AOD users. An American study assessed the readability of materials from a nationwide sample of alcohol and drug abuse treatment programs and reported that the readability level exceeded the average reading ability of adults, reaching a grade level of 11.84 [21]. Similar findings have been reported in studies examining written educational materials on various health topics such as obesity management [22], oral health [23, 24], stroke management [25] and surgery [26]. A recent study on AOD resources specifically designed for Aboriginal and Torres Strait Islander peoples [27] highlighted that the graphical elements, written communications, content and readability differed, and they were not culturally appropriate for all communities. Furthermore, an Australian study among individuals seeking specialised treatment for substance use disorders revealed that a significant proportion of participants engaged in residential substance abuse treatment had low-to-moderate health literacy levels [28]. All the above findings raise concerns, as individuals suffering from AOD disorders often need comprehensive health services. Access to appropriate health resources becomes crucial in mitigating the risk of potential diseases, hospitalisations, mental health challenges and unintentional death [1, 21]. Unsurprisingly, there is a dearth of evidence on the comprehensive evaluation of the content, suitability and readability of educational resources specifically designed for AOD users, as well as their families and caregivers within the NSW healthcare system. Therefore, the objective of this study was to conduct a comprehensive audit to analyse the content, suitability, and readability of government and not-for-profit AOD online resources available in NSW.

## Methods

### Data collection

We conducted a comprehensive desktop search to identify AOD resources available in NSW between July and August 2023. The search aimed to capture a representative sample of online materials, readily available to the public, produced by government and not-for-profit organisations that were based in NSW. The search was initiated using the key terms ‘alcohol’, ‘drugs’ and ‘resources’ in Google, Chrome and organisational websites. This research examined readily accessible and pre-existing materials and did not necessitate ethical clearance [29].

Eligible resources included publicly accessible online materials developed by government or not-for-profit organisations based in NSW. Resources were required to be written in English and intended for individuals using AOD or their families and/or carers. Only materials that contained educational content or harm-reduction information related to AOD were included. We specifically selected resources with a concise length of one to two pages in alignment with recommendations from ‘Simply put: a guide for creating easy-to-understand materials’ developed by The Centers for Disease Control and Prevention. This guide provides evidence-informed strategies to improve the clarity and effectiveness of public health communication, particularly for audiences with varying levels of health literacy. It emphasises the importance of simplifying complex information by focusing on the most essential messages and limiting the number of key ideas per document. Additionally, the guide suggests presenting a single complete concept on one or two facing pages to enhance comprehension and retention [30]. Research conducted by Jansen et al. [31] also indicates that limiting health resources to a maximum of two pages can potentially boost resource comprehension, particularly among individuals with lower health literacy.

Resources were excluded if they originated from outside NSW, were produced by private or commercial entities, lacked relevant AOD content, or exceeded the specified page limit. A complete list of the inclusion and exclusion criteria is provided in Appendix 1.

### Resource appraisal

Each resource was subjected to a thorough appraisal process by two reviewers (AV and AA) based on attributes, visual and written communication, thoroughness and content, and readability. We piloted our data extraction for two resources and then two reviewers (AV and AA) conducted the data extraction across all domains for all resources independently. If there were any disagreements, these were resolved through the involvement of a third reviewer (RA).

### Resource attributes

We used a comprehensive documentation process to analyse the resource characteristics, encompassing essential elements such as resource title, the name of the publishing organisation, and the classification of organisation type into either government or not-for-profit organisation. Additionally, we assessed the primary focus of the resource, specifically whether it addressed alcohol-related topics, drug-related topics, or both. The target audience of each resource was identified (for example: women, young people etc.), and physical attributes were recorded, including the format (such as factsheet, poster, postcard, brochure, or pamphlet) and the number of pages it comprised. Importantly, we also determined whether the authors had explicitly described consumer participation in the development process [32]. Consumer involvement ensures that materials are relevant, accessible, and reflective of real-world needs, particularly for populations with lower health literacy or those facing barriers to engagement [33, 34]. Research has shown that consumer participation contributes to positive outcomes, including improved quality of care, greater treatment adherence, higher levels of satisfaction, and better health outcomes overall [35].

### Thoroughness and content

Previous research conducted by Arora et al. [32] and Amanda et al. [36] guided the development of the content categories and shaped the organisation and assessment of topics. The analysis covered 18 AOD topics across four domains:

1. Substance information and effects –general information; acute effects; chronic effects.
2. Health and social impacts –physical health; mental health; social life; harm to others; pregnancy and breastfeeding.
3. Risk and harm reduction – drug interactions; tolerance and dependence; symptoms of overdose; harm minimisation during overdose; withdrawal symptoms; withdrawal harm minimisation.
4. Support, treatment, and context –detoxification, treatment, and prevention strategies; contact information for support services; legal framework; information for family and caregivers.

### Visual and written communication

We used the Suitability Assessment of Materials (SAM) metric to subjectively analyse visual and written elements of the resources. Doak [37] developed the SAM instrument to systematically evaluate the appropriateness of health information materials for a specific audience in a limited timeframe. It consists of 22 items grouped under six factors—content, literacy demand, graphics, layout and typography, learning stimulation and motivation, and cultural appropriateness—and has been successfully applied in previous studies evaluating health information [37, 38].Each item is scored on an ordinal scale where rating of ‘superior’ earns 2 points, ‘adequate’ earns 1 point, and ‘not suitable’ 0 points [37]. The SAM tool can help to gain a thorough understanding of how the text and graphics of AOD resources affect their suitability for a variety of audiences.

Due to the presence of overlapping criteria with other methodologies used in this study, we only included a few factors from SAM’s criteria. Five factors: purpose, layout factors, typography, types of drawings, and writing style were assessed using the SAM evaluation tool. These factors were categorised as either ‘superior’, ‘adequate’ or ‘not suitable’ based on the predetermined criteria. Specifically, we gave a ‘superior’ rating for ‘purpose’ when the primary aim of the resource was explicitly addressed in the title or introduction. Similarly, in the case of ‘type of illustrations’, a ‘superior’ rating was awarded when the resource contained simple line sketches that the target audience could understand. Furthermore, ‘superior’ typography for optimal readability involves a mix of uppercase and lowercase letters, a minimum type size of 12 points, the strategic use of typographic cues like bold text and colour variations, and avoiding long sections of text in all caps [37].

Dual Code Theory suggests that individuals process and retain information through two interconnected cognitive channels: the verbal system and the visual system [39]. When text and relevant visuals are presented simultaneously, they activate both systems in parallel, enhancing comprehension and memory retention by creating stronger associative links [40]. The principles of Dual Code Theory were utilised to evaluate factors like the relevance of the drawings, ratio of visual elements to written text, and the use of colour supports. The materials were marked ‘Y’ if they fulfilled these criteria [40].

We used Kool’s model for macro-level and micro-level coherence to assess the structure and coherence of the written materials [41]. Text coherence at the micro and macro levels is a significant factor in enhancing text comprehension [41]. At the macro level, coherence refers to the logical arrangement of content across sections, typically supported by the use of clear headings and a consistent flow of ideas. It ensures that the overall structure is easy to follow and that topics are presented in a meaningful sequence. Micro-level coherence, on the other hand, pertains to the connections between sentences and smaller text units, often achieved through cohesive phrasing and appropriate transitions within paragraphs. According to Kool’s framework, one way to strengthen macro-level coherence is through the inclusion of headings and subheadings, while micro-level coherence is enhanced by the use of bulleted text to improve sentence-level clarity [41]. Therefore, we included an analysis of each resource for its use of ‘headings and subheadings’ and ‘percentage of bulleted text’. Resources that fulfilled the criteria were marked ‘Y’ for ‘Yes’ and those that did not meet the criteria were marked ‘-’ for ‘No’.

We also included a subjective screening of each leaflet for jargon usage. A compilation of terms, both identified from a previous study [27] and observed in the leaflets under review, was utilised for this analysis.

### Readability

Readability analysis is an established approach for evaluating the comprehensibility of written information [23, 25, 27, 32]. We used four established metrics, the Flesch Reading Ease Score [42], the Flesch – Kincaid Grade Level (FKGL) [43], the Simplified Measure of Gobbledygook (SMOG) [44] and the Gunning Fog Index (FOG) [45] – to evaluate readability level of the resources. Other researchers have recommended using multiple readability metrics to assess materials for a thorough evaluation [23, 25, 32]. The detailed formulas for the indices are provided in Appendix 2. We chose these matrices because they are commonly employed and are well-suited for evaluating the readability of patient educational material and health information [37].

The Flesch Reading Ease Score considers the average sentence length and the syllable count per word within a passage. This tool gives a score between 0 and 100, and if the score is 100, it means the text is effortless to read by an 11-year-old child or by a person with 5th-grade literacy, while a score of 0 means the text is complex to read and can be read by someone with a college graduate level of literacy (see Appendix 3) [42]. The FKGL, formulated through an assessment of sentence length (in terms of word count) and word complexity (in syllables), originated for United States Armed Forces utilisation. This formula estimates the formal educational level required for comprehending a given text. Longer sentences and words with more syllables indicate a higher level of difficulty [43]. Similarly, the FOG considers average sentence length and percentage of polysyllable words [45]. Additionally, the SMOG grade, acknowledged as the benchmark in health information evaluation, calculates the average grade level required to obtain 100% comprehension by incorporating the square root of words with three or more syllables per three sentences [44, 46].

To assess the readability of the resources utilising the four formulas, we input the title and contents of the AOD resources into an automated web-based tool [47]. Before input, we proofread the text to rectify any spelling errors. Additionally, we transformed the text into plain text, expanding abbreviations such as ‘e.g.’, removing bullet points and eliminating illustrations and text boxes.

### Statistical analysis

We input the data derived from the analysis of visual and written elements, content analysis, physical attributes, as well as readability into Excel sheets and calculated descriptive statistics, including frequency, percentage, and standard deviation. Additionally, we conducted a series of Chi-Square tests to ascertain whether certain resource characteristics (e.g., type of organisation, target audience) predict readability scores. *P* < 0.05 was deemed statistically significant. Statistical Package for Social Science (SPSS) version 22 (SPSS for Windows, SPSS Inc., Chicago, IL, USA) was used for data management and analysis.

## Results

**Fig. 1:**
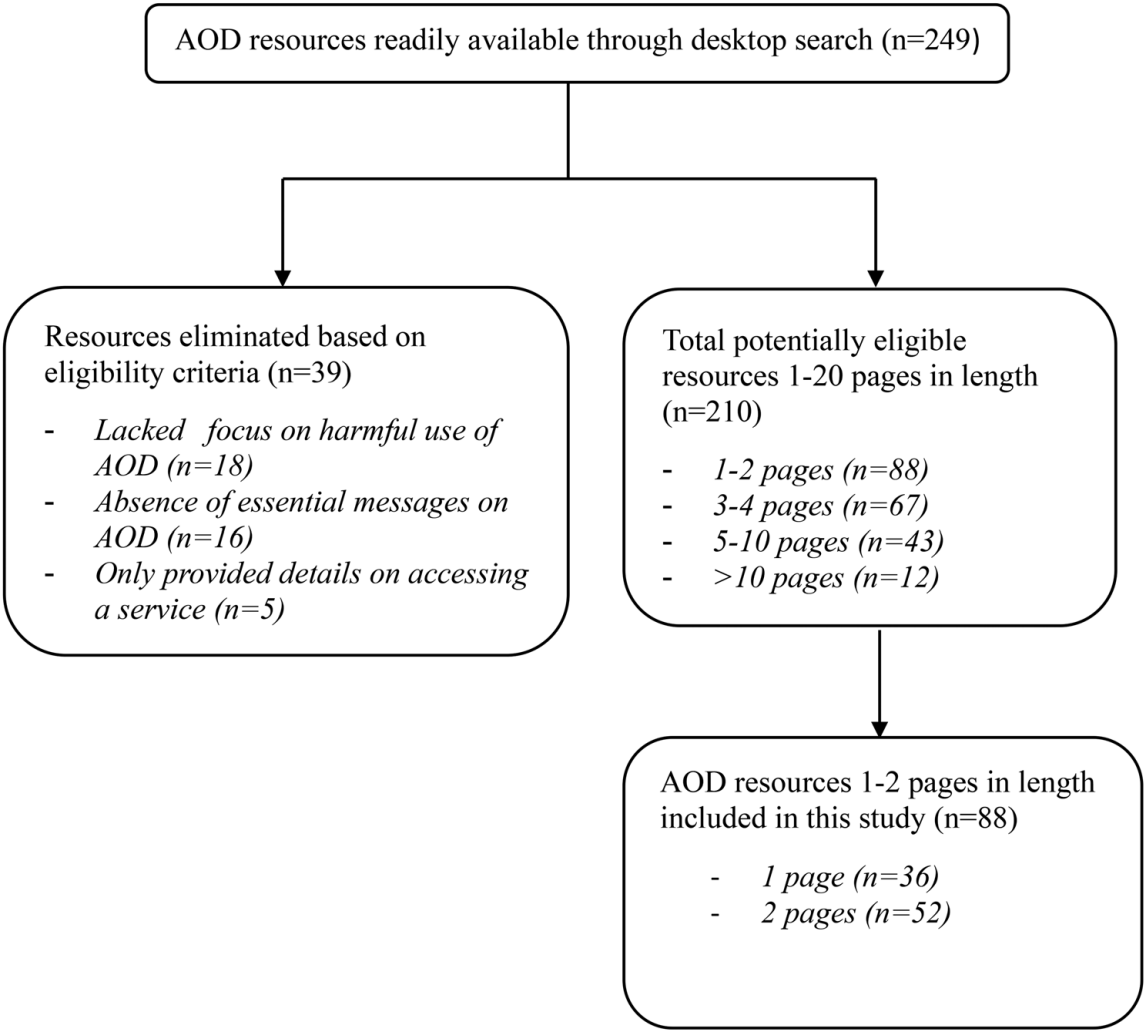
Illustrates the selection of Alcohol and Other Drug (AOD) resources in this study

We procured a total of 249 resources through the online search strategy. Subsequently, we excluded 39 as they did not centre their focus on the problematic utilisation of AOD or did not contain substantial information in this regard. Out of the remaining 210 eligible resources, 88 educational resources were included in this study. Among these 88 resources, 52 (59%) of them were in a two-page format, and the remaining 36 (41%) were single-page publications. The inter-rater agreement between the two reviewers was 98% across all domains (Fig. 1).

### Resource attributes

We identified a total of 88 resources, with 25 of them originating from governmental organisations, 56 from not-for-profit entities and six resources as a collaboration between organisations (Table 1). The primary contributor from government entities was NSW Health, accounting for 19 resources, followed by South Western Sydney Local Health District (SWSLHD) with three resources. Additionally, the NSW Department of Attorney General and Justice and NSW Police Force published one resource each. Not-for-profit organisations also made a considerable impact with Family Drug Support Australia being the most active publisher, contributing 20 resources, followed by the Australian Indigenous Alcohol and Other Drugs Knowledge Centre with 17 resources (refer Table 1 for other organisations). Furthermore, several resources were the result of collaborations between governmental and not-for-profit organisations, including the Australian Government Department of Health and The University of Sydney Matilda Centre, which produced three resources, the Aboriginal Health and Medical Research Council of NSW and Aboriginal Drug and Alcohol Network of NSW collaborating on two resources, and Aboriginal Health and Medical Research Council of NSW in partnership with the Aboriginal Drug and Alcohol Residential Rehab Network contributing one resource.

**Table 1:** A comprehensive overview of the attributes of AOD educational resources

No.	Resource title	Organisation	Organisation type	Target audience	No. of pages	Format	Topic
1	Harm Minimisation and COVID-19	Aboriginal Health and Medical Research Council of NSW, Aboriginal Drug and Alcohol Network of NSW	Government/not-for-profit organisation	Aboriginal communities	1	fact sheet	AOD
2	Magistrates Early Referral into Treatment (MERIT)	NSW Health	Government	AOD users	2	fact sheet	AOD
3	Drug Information: Other Drug Support Services	Family Drug Support Australia	Not-for-profit organisation	AOD users	1	fact sheet	Drugs
4	Cracks in the ice: Information for families and friends	Australian Government Department of Health, The University of Sydney Matilda Centre	Government/not-for-profit organisation	Aboriginal communities	2	brochure	Drugs
5	Key facts: Illicit drug use among Aboriginal and Torres Strait Islander people	Alcohol and Other Drugs Knowledge Centre	Not-for-profit organisation	Aboriginal communities	1	fact sheet	Drugs
6	Drug Information: Ecstasy and related drugs	Family Drug Support Australia	Not-for-profit organisation	AOD users and families	1	fact sheet	Drugs
7	Methamphetamine Use & Blood Borne Virus/Sexually Transmissible Infection (BBV/STI)	Australian Injecting & Illicit Drug Users League (AIVL)	Not-for-profit organisation	AOD users	2	pamphlet	Drugs
8	Inhalants: The Facts	NSW Health	Government	AOD users	2	fact sheet	Drugs
9	Understanding Drug Use: Supporting someone through detox	Family Drug Support Australia	Not-for-profit organisation	Family and caregivers	1	fact sheet	Drugs
10	Drug Information: Ice	Family Drug Support Australia	Not-for-profit organisation	AOD users	1	fact sheet	Drugs
11	Cocaine	Headspace National Youth Mental Health Foundation	Not-for-profit organisation	Young people	2	fact sheet	Drugs
12	Drug Information: Cannabis	Family Drug Support Australia	Not-for-profit organisation	AOD users	1	fact sheet	Drugs
13	Get healthy and reduce your alcohol consumption	NSW Health	Government	AOD users	2	brochure	Alcohol
14	Facts about alcohol	Australian Indigenous Alcohol and Other Drugs Knowledge Centre	Not-for-profit organisation	Aboriginal communities	2	fact sheet	Alcohol
15	Drug Information: Cocaine	Family Drug Support Australia	Not-for-profit organisation	AOD users and families	1	fact sheet	Drugs
16	Amyl Nitrite/Poppers: The facts	NSW Health	Government	AOD users	2	leaflet	Drugs
17	Alcohol: Get the facts	Drink Wise Australia	Not-for-profit organisation	AOD users	2	brochure	Alcohol
18	FAQ: Alcohol, Other Drugs and Mental Health Needs in Young People	Alcohol and Drug Foundation	Not-for-profit organisation	Young people	2	fact sheet	AOD
19	Women who are pregnant or planning a pregnancy should not drink alcohol	Drink Wise Australia	Not-for-profit organisation	AOD users (Women)	1	poster	Alcohol
20	Alcohol and drug treatment for Aboriginal and Torres Strait Islander peoples	Australian Indigenous Alcohol and Other Drugs Knowledge Centre	Not-for-profit organisation	Aboriginal communities	1	fact sheet	AOD
21	Substance misuse – alcohol & other drugs	Lifeline	Not-for-profit organisation	AOD users and families	2	fact sheet	AOD
22	What is FASD?	NSW Health	Government	Aboriginal communities (young people)	2	postcard	Alcohol
23	Breaking the Ice in our Community: Harm reduction and methamphetamine use	NSW Health and Australian Drug Foundation	Government/not-for-profit organisation	AOD users	2	fact sheet	Drugs
24	Methamphetamine use among Aboriginal and Torres Strait Islander people	Alcohol and Other Drugs Knowledge Centre	Not-for-profit organisation	Aboriginal communities	1	fact sheet	Drugs
25	Alcohol and pregnancy	Drink Wise Australia	Not-for-profit organisation	AOD users (Women)	2	brochure	Alcohol
26	Facts about Ganja	Australian Indigenous Alcohol and Other Drugs Knowledge Centre	Not-for-profit organisation	Aboriginal communities	2	fact sheet	Drugs
27	Drug Information: Methadone	Family Drug Support Australia	Not-for-profit organisation	AOD users and families	1	fact sheet	Drugs
28	The Substance Use in Pregnancy Service (SUPPS)	NSW Health SWSLHD	Government	AOD users (Women)	2	pamphlet	AOD
29	Get healthy and reduce your alcohol consumption	NSW Health	Government	AOD users	2	postcard	Alcohol
30	Stay strong and healthy: It’s worth it	NSW Health	Government	Aboriginal communities	1	poster	Alcohol
31	MERIT Magistrates Early Referral into Treatment	NSW Health SWSLHD	Government	AOD users	2	pamphlet	Drugs
32	Methamphetamine Use & Blood Borne Virus/Sexually Transmissible Infection (BBV/STI)	Australian Injecting & Illicit Drug Users League (AIVL)	Not-for-profit organisation	AOD users	1	poster	Drugs
33	Facts about petrol, paint, and other inhalants	Alcohol and Other Drugs Knowledge Centre	Not-for-profit organisation	Aboriginal communities	2	fact sheet	Drugs
34	Stay strong and healthy its worth it	NSW Health	Government	Aboriginal communities(women)	2	postcard	Alcohol
35	Facts about prescription opioids	Australian Indigenous Alcohol and Other Drugs Knowledge Centre	Not-for-profit organisation	Aboriginal communities	2	fact sheet	Drugs
36	Drug Information: Hallucinogens	Family Drug Support Australia	Not-for-profit organisation	AOD users and families	1	fact sheet	Drugs
37	New buprenorphine (Bupe) depot products	Australian Injecting & Illicit Drug Users League (AIVL)	Not-for-profit organisation	AOD users	1	poster	Drugs
38	Ketamine: The Facts	NSW Health	Government	AOD users	2	fact sheet	Drugs
39	Medications	NUAA (NSW Users & AIDS Association)	Not-for-profit organisation	AOD users	2	fact sheet	Drugs
40	Drug Information: Inhalants	Family Drug Support Australia	Not-for-profit organisation	AOD users and families	1	fact sheet	Drugs
41	Recovery and isolation	Aboriginal Health and Medical Research Council of NSW, Aboriginal Drug and Alcohol Network of NSW	Government/not-for-profit organisation	Aboriginal communities	2	fact sheet	AOD
42	Kava use among Aboriginal and Torres Strait Islander people	Alcohol and Other Drugs Knowledge Centre	Not-for-profit organisation	Aboriginal communities	1	fact sheet	Drugs
43	Drug Information: Amphetamine	Family Drug Support Australia	Not-for-profit organisation	AOD users and families	1	fact sheet	Drugs
44	A guide to the Drug Court of New South Wales	NSW Department of Attorney General & Justice	Government	AOD users	2	pamphlet	Drugs
45	Facts about ice	Australian Indigenous Alcohol and Other Drugs Knowledge Centre	Not-for-profit organisation	Aboriginal communities	2	fact sheet	Drugs
46	Facts About Benzodiazepines	Australian Indigenous Alcohol and Other Drugs Knowledge Centre	Not-for-profit organisation	Aboriginal communities	2	fact sheet	Drugs
47	Reducing the risk of workplace alcohol and other drug problems	Alcohol and Drug Foundation	Not-for-profit organisation	AOD users	2	fact sheet	AOD
48	How does alcohol affect mental health?	Headspace National Youth Mental Health Foundation	Not-for-profit organisation	Young people	2	fact sheet	Alcohol
49	Drug Information: Benzodiazepines	Family Drug Support Australia	Not-for-profit organisation	AOD users and families	1	fact sheet	Drugs
50	Harm reduction for families	Family Drug Support Australia	Not-for-profit organisation	AOD users and families	1	fact sheet	Drugs
51	How to recognise an overdose	NUAA (NSW Users & AIDS Association)	Not-for-profit organisation	Family and caregivers	1	poster	Drugs
52	Ecstasy	Headspace National Youth Mental Health Foundation	Not-for-profit organisation	Young people	2	fact sheet	Drugs
53	Drug Information: Heroin	Family Drug Support Australia	Not-for-profit organisation	AOD users and families	1	fact sheet	Drugs
54	Driving Safety and Medicines Patient Information	NSW Health	Government	AOD users	2	brochure	Drugs
55	Facts about new psychoactive substances	Australian Indigenous Alcohol and Other Drugs Knowledge Centre	Not-for-profit organisation	Aboriginal communities	2	fact sheet	Drugs
56	Grog is no good for our babies	NSW Health	Government	Aboriginal communities	1	poster	Alcohol
57	Reducing the health risks of drinking alcohol	Alcohol and Drug Foundation	Not-for-profit organisation	AOD users	2	fact sheet	Alcohol
58	Harm Reduction: Communicating with the drug user	Family Drug Support Australia	Not-for-profit organisation	Family and caregivers	1	fact sheet	Drugs
59	Facts about ecstasy	Australian Indigenous Alcohol and Other Drugs Knowledge Centre	Not-for-profit organisation	Aboriginal communities	2	fact sheet	Drugs
60	Infectious Disease Prevention: safe injecting and safe sex practices	Family Drug Support Australia	Not-for-profit organisation	AOD users and families	1	fact sheet	Drugs
61	Drug Information: Alcohol	Family Drug Support Australia	Not-for-profit organisation	AOD users and families	1	fact sheet	Alcohol
62	Drug Information: Steroids	Family Drug Support Australia	Not-for-profit organisation	AOD users	1	fact sheet	Drugs
63	Drug Information: Other Pharmacotherapies	Family Drug Support Australia	Not-for-profit organisation	AOD users and families	1	fact sheet	Drugs
64	Kids and alcohol don’t mix	Drink Wise Australia	Not-for-profit organisation	Family and caregivers	2	brochure	Alcohol
65	Nitrous Oxide: The Facts	NSW Health	Government	AOD users	2	fact sheet	Drugs
66	New buprenorphine (Bupe) depot products	Australian Injecting & Illicit Drug Users League (AIVL)	Not-for-profit organisation	AOD users	2	pamphlet	Drugs
67	Facts about kava	Australian Indigenous Alcohol and Other Drugs Knowledge Centre	Not-for-profit organisation	Aboriginal communities	2	fact sheet	Drugs
68	Synthetic Drugs: The Facts	NSW Health	Government	AOD users	2	fact sheet	Drugs
69	Ecstasy: The Facts	NSW Health	Government	AOD users	2	fact sheet	Drugs
70	Key facts: Volatile substance use (VSU) among Aboriginal and Torres Strait Islander people	Alcohol and Other Drugs Knowledge Centre	Not-for-profit organisation	Aboriginal communities	1	fact sheet	Drugs
71	What is FASD?	NSW Health	Government	Aboriginal communities	1	poster	Alcohol
72	Detox, rehab, and COVID-19	Aboriginal Health and Medical Research Council of NSW, Aboriginal Drug and Alcohol Residential Rehab Network	Not-for-profit organisation	Aboriginal communities	2	fact sheet	AOD
73	Not Our Way: Are you standing on thin ice?	NSW Police Force	Government	Aboriginal communities	2	postcard	Drugs
74	Alcohol and other drugs in the Workplace	Alcohol and Drug Foundation	Not-for-profit organisation	AOD users	2	fact sheet	AOD
75	Cracks in the ice: Information for the community	Australian Government Department of Health, The University of Sydney Matilda Centre	Government/not-for-profit organisation	Aboriginal communities	2	brochure	Drugs
76	Key facts: Alcohol use among Aboriginal and Torres Strait Islander people	Alcohol and Other Drugs Knowledge Centre	Not-for-profit organisation	Aboriginal communities	1	fact sheet	Alcohol
77	Harm reduction for the drug user	Family Drug Support Australia	Not-for-profit organisation	Family and caregivers	1	fact sheet	Drugs
78	Facts about heroin	Alcohol and Other Drugs Knowledge Centre	Not-for-profit organisation	Aboriginal communities	2	fact sheet	Drugs
79	Synthetic Drugs	Family Drug Support Australia	Not-for-profit organisation	AOD users and families	1	fact sheet	Drugs
80	Do you regularly take opioids? Are you aware of your risk of overdose?	NSW Health	Government	AOD users	2	postcard	Drugs
81	How to respond to an overdose	NUAA (NSW Users & AIDS Association)	Not-for-profit organisation	Family and caregivers	1	poster	Drugs
82	Information for Patients: Driving Safety and Medicines	NSW Health	Government	AOD users	2	fact sheet	Drugs
83	Fatal Alcohol Spectrum Disorder (FASD) among Aboriginal and Torres Strait Islander people	Alcohol and Other Drugs Knowledge Centre	Not-for-profit organisation	Aboriginal communities	1	fact sheet	Alcohol
84	Do you support someone who uses or is dependent on alcohol or drugs?	NSW Health	Government	Family and caregivers	2	brochure	AOD
85	A guide for counting your drinks	Alcohol and Drug Foundation	Not-for-profit organisation	AOD users	2	fact sheet	Alcohol
86	Drug Health Services	NSW Health SWSLHD	Government	AOD users	2	pamphlet	AOD
87	Cracks in the ice: Information for staying safe	Australian Government Department of Health, The University of Sydney Matilda Centre	Government/not-for-profit organisation	Aboriginal communities	2	brochure	Drugs
88	Steroids: The facts	NSW Health	Government	AOD users	2	leaflet	Drugs

*NSW – New South Wales, SWSLHD- South Western Sydney Local Health District

Most of the resources were factsheets (*n* = 58), followed by brochures (*n* = 9), posters (*n* = 8), pamphlets (*n* = 6), postcards (*n* = 5), and finally, leaflets (*n* = 2).

Our analysis of the target populations of AOD resources revealed that the majority of the resources (n=31) were designed for AOD users, with a specific focus on women for three resources. Aboriginal and Torres Strait Islander people were a key population group for 27 resources, including specialised content for both women and young people. Additionally, 14 resources were published for the support of AOD users and families, a further seven were targeted towards families and caregivers, and four were targeted towards young people.

In three resources, ‘Fetal alcohol spectrum disorder (FASD) among Aboriginal and Torres Strait Islander people’, ‘Kava use among Aboriginal and Torres Strait Islander people’, and ‘Methamphetamine use among Aboriginal and Torres Strait Islander people’, consumer participation was evident in the creation of artwork during the resource development process. The development of the other resources lacked consumer involvement.

### Thoroughness and content

Table 2 presents the topics and subtopics addressed in the 88 unique AOD resources. Most resources (*n* = 58; 66%) focused on drug-related content. In contrast, 20% of the resources (*n* = 18) were dedicated to alcohol-related topics, while 14% (*n* = 12) covered both alcohol and other drugs.

**Table 2:** Overview of content analysis of AOD educational resources

No.	Substance information and effects			Impact of AOD substances					Drug interactions	Tolerance and dependence	Overdose		Withdrawal		Detoxification, treatment, and prevention strategies	Contact information for support services	Legal framework	Information for family and caregivers
	General information	Acute effects	Chronic effects	Physical health	Mental health	Social impacts	Harm to others	Pregnancy and breastfeeding			Symptoms	Harm minimisation	Symptoms	Harm minimisation				
1	Y	-	Y	-	-	-	-	Y	-	-	-	-	-	-	Y	-	-	-
2	Y	Y	Y	Y	Y	-	Y	Y	-	Y	-	-	Y	-	-	-	Y	-
3	Y	Y	Y	Y	Y	-	-	Y	-	-	-	-	-	-	Y	-	-	-
4	Y	Y	Y	Y	-	-	-	-	-	Y	-	-	-	-	-	Y	Y	Y
5	Y	-	-	Y	Y	Y	Y	-	-	-	-	-	-	-	-	-	-	-
6	Y	-	Y	Y	Y	Y	Y	-	-	Y	-	-	-	-	-	Y	Y	Y
7	-	Y	-	-	-	-	-	-	-	-	Y	-	-	-	-	-	-	-
8	Y	Y	Y	Y	Y	-	-	-	Y	-	-	-	-	-	-	-	-	-
9	Y	Y	Y	Y	Y	Y	Y	-	Y	Y	Y	Y	Y	Y	Y	Y	Y	Y
10	Y	Y	Y	Y	Y	Y	Y	-	Y	Y	Y	Y	Y	Y	Y	Y	Y	Y
11	Y	Y	Y	Y	Y	Y	-	-	-	-	-	-	-	-	Y	Y	-	Y
12	-	-	-	-	-	-	-	-	-	Y	-	-	-	-	-	Y	-	Y
13	Y	Y	-	Y	-	Y	Y	-	-	Y	Y	Y	Y	Y	Y	Y	-	-
14	-	-	-	-	-	-	-	-	-	-	-	-	-	-	Y	Y	Y	-
15	-	Y	-	Y	-	-	Y	Y	Y	Y	Y	Y	-	-	Y	-	-	Y
16	Y	Y	-	Y	-	-	Y	-	-	-	-	-	-	-	Y	-	-	-
17	Y	-	-	-	-	-	-	-	-	-	-	-	-	-	Y	Y	-	Y
18	Y	-	-	-	-	-	-	Y	-	-	-	-	-	-	Y	-	-	-
19	Y	Y	Y	Y	Y	Y	-	-	-	Y	Y	Y	Y	-	Y	Y	Y	Y
20	-	-	-	-	-	-	-	-	-	-	-	Y	-	-	-	-	-	Y
21	-	Y	-	Y	Y	-	Y	-	-	-	-	-	-	-	-	Y	-	Y
22	-	-	-	-	-	Y	-	-	-	-	-	-	-	-	-	-	-	Y
23	-	-	-	-	-	-	-	-	-	-	-	-	-	-	Y	Y	-	-
24	Y	Y	Y	Y	Y	Y	-	-	Y	Y	Y	-	-	-	-	Y	-	-
25	Y	-	Y	Y	Y	Y	-	-	-	-	-	-	-	-	-	Y	-	Y
26	Y	Y	-	Y	-	-	-	-	-	-	-	-	-	-	-	-	-	-
27	Y	Y	-	Y	Y	-	Y	-	-	-	-	-	-	-	Y	Y	-	-
28	Y	Y	Y	Y	Y	-	Y	-	-	-	-	-	-	-	-	Y	Y	Y
29	Y	Y	-	Y	Y	Y	Y	-	-	-	-	-	-	-	Y	Y	-	-
30	Y	-	-	Y	-	-	-	-	-	-	-	Y	Y	Y	Y	Y	-	-
31	Y	-	-	-	-	-	-	-	-	-	-	-	-	-	-	Y	-	-
32	Y	Y	Y	Y	-	Y	-	-	Y	-	-	-	-	-	-	-	Y	-
33	Y	Y	Y	Y	Y	-	Y	Y	Y	Y	-	-	Y	-	Y	Y	Y	Y
34	Y	Y	Y	Y	Y	-	Y	Y	Y	-	-	-	-	-	-	Y	Y	Y
35	-	Y	Y	Y	Y	-	-	Y	Y	Y	Y	Y	-	-	-	Y	Y	Y
36	Y	Y	Y	Y	Y	-	Y	Y	Y	Y	Y	Y	-	-	-	Y	Y	Y
37	-	-	-	-	-	-	-	-	-	-	-	-	-	-	Y	Y	-	-
38	Y	Y	Y	Y	Y	-	Y	Y	Y	Y	-	-	Y	-	-	Y	Y	Y
39	Y	Y	Y	Y	Y	Y	-	-	-	-	-	-	-	-	Y	-	-	-
40	-	Y	Y	Y	Y	Y	Y	-	-	-	-	-	-	-	-	Y	-	Y
41	Y	-	Y	Y	Y	-	-	-	-	-	-	-	-	-	Y	-	-	-
42	Y	Y	Y	Y	Y	-	-	Y	-	Y	-	Y	Y	-	-	Y	-	Y
43	Y	Y	-	Y	Y	-	Y	-	-	-	Y	Y	Y	-	-	Y	Y	Y
44	Y	-	-	-	-	-	-	-	-	-	-	-	-	-	-	Y	-	-
45	Y	Y	Y	Y	Y	Y	Y	Y	-	-	Y	Y	Y	Y	Y	-	-	Y
46	-	-	-	-	-	-	-	Y	-	-	-	-	-	-	Y	-	-	-
47	Y	Y	Y	Y	Y	-	-	-	Y	-	Y	-	-	-	Y	Y	Y	Y
48	Y	Y	-	Y	-	-	Y	-	-	-	-	-	-	-	-	Y	-	-
49	Y	Y	Y	Y	Y	-	Y	Y	Y	Y	Y	Y	-	-	-	Y	Y	Y
50	Y	Y	Y	Y	Y	-	-	-	-	Y	Y	Y	Y	-	-	Y	-	-
51	Y	-	-	-	-	-	-	-	-	-	-	-	-	-	Y	-	-	-
52	Y	Y	Y	Y	Y	Y	-	-	-	-	-	-	Y	-	Y	-	-	Y
53	-	-	Y	-	-	-	-	Y	-	-	-	-	-	-	-	-	-	-
54	-	-	-	-	-	-	-	Y	-	-	-	-	-	-	Y	-	-	-
55	Y	-	-	-	-	-	-	Y	Y	Y	Y	Y	-	Y	Y	Y	-	Y
56	Y	Y	Y	Y	Y	Y	-	Y	Y	Y	Y	-	Y	-	Y	-	-	-
57	Y	Y	Y	Y	Y	Y	Y	-	Y	Y	Y	Y	Y	Y	Y	Y	Y	Y
58	Y	Y	Y	Y	Y	Y	Y	Y	-	-	-	-	-	-	Y	-	-	-
59	Y	Y	-	Y	Y	Y	Y	Y	-	-	-	-	-	-	-	Y	-	-
60	Y	Y	-	Y	Y	-	Y	-	-	-	-	-	-	-	Y	Y	-	-
61	-	-	-	-	-	-	-	Y	-	-	-	-	-	-	-	-	-	-
62	-	-	-	-	-	-	-	-	-	Y	-	-	-	-	Y	-	-	-
63	Y	-	-	-	-	-	-	-	-	-	-	Y	-	-	Y	Y	-	-
64	Y	Y	Y	-	Y	-	-	-	-	-	-	-	-	-	Y	Y	-	Y
65	Y	Y	Y	Y	Y	Y	-	-	-	Y	Y	Y	Y	-	Y	Y	-	Y
66	-	-	-	-	-	-	-	-	-	-	-	-	-	-	Y	Y	-	-
67	-	-	-	-	-	-	-	-	-	-	-	-	-	-	Y	Y	Y	-
68	-	-	-	-	-	-	-	-	-	-	-	-	-	-	Y	Y	-	-
69	Y	Y	Y	Y	Y	Y	Y	Y	Y	Y	Y	-	Y	-	Y	Y	-	Y
70	Y	Y	-	Y	Y	Y	Y	-	-	-	-	-	-	-	-	Y	Y	-
71	Y	Y	Y	Y	Y	Y	Y	-	Y	Y	Y	Y	Y	-	Y	Y	-	Y
72	Y	Y	Y	Y	Y	-	-	-	Y	-	Y	-	Y	-	-	Y	-	-
73	-	-	Y	Y	-	-	Y	-	-	-	-	-	-	-	Y	-	-	-
74	Y	Y	Y	Y	-	-	-	-	-	Y	Y	Y	Y	Y	-	Y	-	Y
75	Y	Y	Y	Y	Y	-	Y	-	Y	Y	-	-	-	-	Y	-	Y	-
76	-	-	-	-	-	-	-	-	-	-	-	-	-	-	Y	Y	Y	-
77	Y	Y	Y	Y	Y	-	Y	-	-	-	-	-	-	-	-	Y	Y	-
78	Y	Y	Y	Y	Y	-	-	Y	-	Y	-	-	Y	-	-	Y	-	-
79	Y	Y	Y	Y	-	Y	Y	-	-	-	-	-	-	-	-	Y	-	-
80	Y	Y	Y	Y	Y	-	Y	-	-	-	Y	Y	Y	-	-	Y	Y	Y
81	Y	Y	Y	Y	Y	Y	Y	Y	Y	Y	-	-	Y	Y	-	Y	Y	Y
82	Y	Y	Y	Y	Y	Y	Y	-	Y	Y	Y	Y	Y	Y	Y	Y	Y	Y
83	Y	-	-	-	-	Y	-	-	-	-	-	-	-	-	-	Y	Y	-
84	Y	Y	Y	Y	Y	Y	Y	-	Y	Y	-	-	-	-	Y	Y	-	Y
85	-	-	-	-	-	-	-	Y	-	-	-	-	-	-	-	-	-	-
86	-	-	-	-	-	-	-	-	-	-	-	-	-	-	Y	Y	-	-
87	Y	Y	Y	Y	Y	Y	-	-	-	Y	-	-	Y	Y	-	Y	-	-
88	Y	Y	Y	Y	-	-	-	Y	Y	Y	Y	Y	Y	Y	Y	Y	Y	Y

*’ Y’ represents yes and ‘- ‘represents No

There was a strong focus on the effects of alcohol consumption during pregnancy and breastfeeding, primarily addressing alcohol as the main topic. Particularly, two resources titled ‘Grog is no good for our babies’ and ‘Stay strong and healthy: It’s worth it’ focused exclusively on the impact of alcohol use during pregnancy. On the other hand, symptoms and harm minimisation in overdose and withdrawal, tolerance and dependence, drug interactions and legal frameworks were discussed in resources with a primary focus on drugs. Four factsheets titled ‘Drug information: Benzodiazepines’, ‘Drug information: Heroin’, ‘Drug information: Amphetamine’, and ‘Drug information: Cocaine’ achieved a significant and thorough coverage by addressing 95% (n = 17) of the AOD topics. These four resources delved into all topics except the impact of drug use during pregnancy and breastfeeding.

In summary, a significant proportion, i.e., 39 resources (accounting for 44% of resources), provided a comprehensive coverage of nine or more topics, with eight resources impressively covering more than 15 topics. However, a notable subset of 32 resources focused on a limited range, addressing only five or fewer topics.

### Visual and written communication

Table 3 shows the findings of the 13 factors related to the visual aspects and written elements of the resources. Our analysis showed that nearly all resources (97%, *n* = 85) had a ‘superior’ rating for the use of headings. Additionally, most resources (90%) had used subheadings.

**Table 3:** Visual aspects and written elements of the AOD educational resources in NSW

No.	Purpose	Subheadings	Layout factors	Typography	bulleted text (%)	Visual to written text (%)	Writing style	Active Voice	Medical jargons	Type of drawing	Relevance of drawings	Infographics	Use of colour supports
1	Superior	Y	Adequate	Adequate	70	0	superior	Y	Y	-	-	-	Y
2	Superior	-	Adequate	Adequate	0	80	Adequate	Y	-	-	-	-	Y
3	Superior	Y	Adequate	Adequate	20	20	Adequate	-	Y	Superior	Y	Y	Y
4	Superior	Y	Adequate	Adequate	0	50	Not suitable	-	-	Superior	Y	Y	Y
5	Superior	Y	Superior	Adequate	0	80	Not suitable	-	-	Superior	Y	Y	Y
6	Superior	Y	Superior	Adequate	40	70	Adequate	-	-	Superior	Y	Y	Y
7	Superior	Y	Adequate	Adequate	0	80	Not suitable	-	Y	Superior	Y	Y	Y
8	Superior	Y	Adequate	Adequate	0	85	Not suitable	-	Y	Superior	Y	Y	Y
9	Superior	Y	Adequate	Adequate	75	25	Adequate	Y	-	-	-	-	Y
10	Superior	Y	Adequate	Adequate	60	20	Adequate	Y	-	-	-	-	Y
11	Superior	Y	Adequate	Adequate	40	30	Adequate	Y	Y	-	-	-	Y
12	Superior	Y	Adequate	Adequate	60	20	Adequate	Y	Y	-	-	-	Y
13	Superior	Y	Adequate	Adequate	50	20	Adequate	Y	Y	-	-	-	Y
14	Superior	Y	Adequate	Adequate	40	25	Adequate	Y	Y	-	-	-	Y
15	Superior	Y	Adequate	Adequate	40	30	Adequate	Y	Y	-	-	-	Y
16	Superior	Y	Adequate	Adequate	10	20	Adequate	Y	Y	-	-	-	Y
17	Superior	Y	Adequate	Adequate	25	0	Adequate	Y	Y	-	-	-	Y
18	Superior	Y	Adequate	Adequate	0	40	Adequate	Y	Y	-	-	-	Y
19	Superior	Y	Adequate	Adequate	25	40	Adequate	Y	Y	-	-	-	Y
20	Adequate	Y	Adequate	Adequate	0	20	Adequate	Y	Y	-	-	-	Y
21	Superior	Y	Superior	Adequate	30	10	Adequate	Y	Y	-	-	-	Y
22	Superior	Y	Superior	Adequate	25	10	Adequate	Y	Y	-	-	-	Y
23	Superior	Y	Superior	Adequate	10	0	superior	Y	Y	-	-	-	Y
24	Superior	Y	Adequate	Adequate	40	20	Adequate	Y	Y	-	-	-	-
25	Superior	Y	Adequate	Adequate	60	20	Adequate	Y	Y	-	-	-	Y
26	Superior	Y	Adequate	Adequate	10	20	Adequate	Y	-	Adequate	Y	-	Y
27	Adequate	Y	Adequate	Adequate	5	10	Adequate	Y	Y	-	-	-	Y
28	Adequate	Y	Adequate	Adequate	25	10	Adequate	Y	Y	-	-	-	Y
29	Adequate	Y	Superior	Adequate	25	10	Adequate	Y	Y	-	-	-	Y
30	Adequate	Y	Adequate	Adequate	30	10	Adequate	Y	Y	-	-	-	Y
31	Superior	-	Superior	Adequate	0	60	Superior	Y	-	-	-	-	Y
32	Adequate	-	Superior	Adequate	0	60	Superior	Y	-	-	-	-	Y
33	Superior	-	Superior	Adequate	0	65	Superior	Y	-	-	-	-	Y
34	Superior	-	Superior	Adequate	0	50	superior	Y	-	Adequate	Y	Y	Y
35	Superior	-	Superior	Adequate	0	40	Superior	Y	Y	Superior	Y	-	Y
36	Superior	Y	Adequate	Adequate	10	0	Superior	Y	Y	Superior	Y	Y	Y
37	Adequate	Y	Adequate	Adequate	75	20	Superior	Y	Y	-	-	Y	Y
38	Adequate	Y	Adequate	Adequate	0	25	Adequate	Y	Y	-	-	Y	Y
39	Adequate	Y	Superior	Adequate	70	0	Adequate	Y	Y	-	-	-	Y
40	Superior	-	Adequate	Adequate	0	0	Superior	Y	Y	-	-	-	Y
41	Superior	Y	Adequate	Adequate	30	0	Adequate	Y	-	-	-	-	Y
42	Adequate	Y	Adequate	Adequate	20	0	Adequate	Y	-	-	-	-	-
43	Superior	Y	Superior	Adequate	0	25	superior	Y	Y	Superior	Y	-	-
44	Superior	Y	Superior	Adequate	40	50	Adequate	Y	-	Superior	Y	-	Y
45	Superior	Y	Adequate	Adequate	85	0	Not suitable	Y	Y	-	-	Y	Y
46	Superior	Y	Adequate	Adequate	70	0	Adequate	Y	Y	-	-	Y	Y
47	Superior	Y	Adequate	Adequate	35	30	Superior	Y	Y	Superior	Y	Y	Y
48	Superior	Y	Adequate	Adequate	60	10	Adequate	Y	Y	Adequate	Y	Y	Y
49	Superior	Y	Adequate	Adequate	10	25	Superior	Y	Y	Superior	Y	Y	Y
50	Superior	Y	Adequate	Adequate	80	0	Adequate	-	-	-	-	-	Y
51	Superior	Y	Adequate	Adequate	75	0	Adequate	Y	-	-	-	-	Y
52	Superior	Y	Adequate	Adequate	0	30	superior	Y	-	-	-	-	Y
53	Superior	Y	Superior	Adequate	0	30	Adequate	Y	Y	-	-	-	-
54	Superior	Y	Adequate	Adequate	20	60	Adequate	Y	Y	-	-	-	-
55	Superior	Y	Superior	Adequate	0	50	superior	Y	Y	Superior	Y	Y	Y
56	Superior	Y	Superior	Adequate	20	0	superior	Y	-	-	-	Y	Y
57	Superior	Y	Superior	Adequate	0	50	superior	Y	Y	Superior	Y	Y	Y
58	Adequate	Y	Adequate	Adequate	70	20	superior	Y	Y	-	-	-	-
59	Adequate	Y	Adequate	Adequate	30	0	Not suitable	-	Y	-	-	-	Y
60	Superior	Y	Adequate	Adequate	50	10	superior	Y	-	Adequate	Y	-	-
61	Superior	Y	Adequate	Adequate	25	0	Not suitable	-	-	-	-	-	-
62	Adequate	-	Adequate	not suitable	10	40	Adequate	Y	-	Adequate	Y	Y	Y
63	Superior	Y	superior	Adequate	25	0	superior	Y	Y	-	-	-	-
64	Superior	Y	superior	Adequate	50	0	Adequate	Y	Y	-	-	-	-
65	Superior	Y	superior	Adequate	25	0	Adequate	Y	Y	-	-	-	-
66	Superior	Y	superior	Adequate	60	0	superior	Y	Y	-	-	Y	Y
67	Superior	Y	Adequate	Adequate	20	10	superior	Y	Y	-	-	Y	Y
68	Superior	Y	superior	Adequate	10	0	superior	Y	Y	-	-	-	-
69	Superior	Y	superior	Adequate	20	0	superior	Y	Y	-	-	-	Y
70	Superior	Y	superior	Adequate	20	0	superior	Y	Y	-	-	-	Y
71	Superior	Y	Adequate	Adequate	40	50	Adequate	Y	-	Superior	Y	Y	-
72	Superior	Y	Adequate	Adequate	50	50	Superior	Y	-	-	-	-	Y
73	Superior	Y	Adequate	Adequate	20	20	superior	Y	-	Adequate	Y	-	Y
74	Superior	Y	Adequate	Adequate	15	50	Adequate	Y	Y	-	-	-	Y
75	Superior	Y	Adequate	not suitable	0	10	Adequate	Y	Y	-	-	-	Y
76	Adequate	Y	Adequate	Adequate	60	0	not suitable	-	Y	-	-	-	-
77	Superior	-	Adequate	Adequate	70	10	Adequate	Y	Y	-	-	-	-
78	Adequate	Y	Superior	Adequate	0	30	Adequate	Y	-	Superior	Y	Y	Y
79	Adequate	Y	Adequate	Adequate	50	10	not suitable	Y	Y	-	-	-	Y
80	Superior	Y	Adequate	Adequate	25	0	Adequate	Y	Y	-	-	Y	-
81	Superior	Y	Adequate	Adequate	50	0	Adequate	Y	Y	-	-	-	-
82	Superior	Y	Adequate	Adequate	50	0	Adequate	Y	Y	-	-	-	-
83	Superior	Y	Adequate	Adequate	70	0	Adequate	Y	Y	-	-	-	-
84	Superior	Y	Adequate	Adequate	30	0	Adequate	Y	Y	-	-	-	-
85	Superior	Y	Adequate	Adequate	80	0	Adequate	Y	Y	-	-	Y	-
86	Superior	Y	Adequate	Adequate	60	0	Adequate	Y	Y	-	-	-	-
87	Superior	Y	Adequate	Adequate	80	0	Adequate	Y	Y	-	-	-	-
88	Superior	Y	Adequate	Adequate	50	0	Adequate	Y	Y	-	-	-	-

*‎ “Y”-Yes

*Layout and typography* While considering layout and typography, more than one-quarter (28%) of the resources had ‘superior’ scoring for layout, and the rest obtained ‘adequate’ ratings (72%) as they fulfilled at least three of the conditions in the SAM criteria. Consistency in the arrangement and order of information and effective use of contrasting colours were observed in these resources. Conversely, while 97% of the resources obtained an ‘adequate’ rating in typography, none achieved ‘superior’ ratings, as most resources lacked typographic cues, consistency in front size (> 12) and font type. For example, factsheets like ‘Kava use among Aboriginal and Torres Strait Islander people’ and ‘Methamphetamine use among Aboriginal and Torres Strait Islander people’ obtained ‘superior’ ratings in layout and ‘adequate’ ratings for typography. An exemplary resource demonstrating ‘superior’ layout quality is ‘Kava use among Aboriginal and Torres Strait Islander people’. This resource effectively incorporates relevant illustrations, employs visual cueing elements, utilises contrasting colours, maintains organised writing, and ensures layout consistency. However, it falls short in font consistency, occasionally deviating from SAM criteria by using a font size of less than 12, resulting in an ‘adequate’ rating for typography.

*Type of Drawings* Only 18% of the resources received ‘superior’ ratings for illustrations, while 5.6% (*n* = 5) achieved ‘adequate’ ratings. Overall, the performance of resources in terms of drawings was lacking, with 71% (*n* = 67) not containing any simple, easy-to-understand line drawings. We found similar results in the use of infographics, with 70% of resources lacking tables or charts. Moreover, one-quarter of the resources (*n* = 21) did not feature bulleted text.

*Writing style* We awarded ‘superior’ ratings for writing style to 24 resources, reflecting conversational style and consistent use of active voice (*n* = 78). Additionally, we deemed 50 resources as ‘adequate’ in this aspect. However, we identified the tendency to incorporate complex sentences with a higher degree of embedded information as a prominent limitation.

*Medical jargon* In the examination of medical jargon within the AOD resources, we identified a comprehensive list of 22 commonly used professional terms (Table 4). Notable among these terms were ‘symptoms’, ‘detoxification’, ‘overdose’, ‘substances’ and ‘illicit drugs’, which were repeatedly used among the resources. In total, we found 63 resources that contained at least one medical term without explanation, underscoring the widespread use of medical terminology in these resources.

**Table 4:** List of medical jargons

Analgesics
Cognitive
Cope
Dependency
Detox
Disability
Illicit drugs
Inhalants
Isolation
Psychosis
Overdose
Relapse
Sedatives
Stimulants
Substances
Symptoms
Synthetic
Tolerance
Toxicity /intoxication.
Withdrawal
Sterile
Chronic

### Readability

**Fig. 2:**
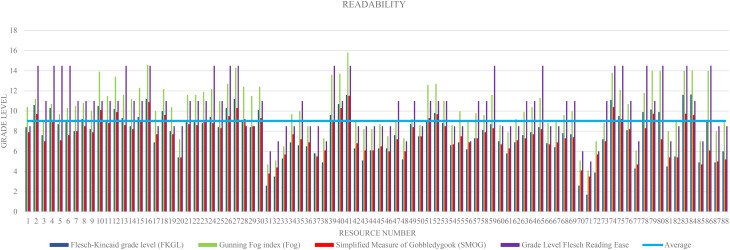
Describes the reading grade levels of the various AOD resources included in this study

The average reading grade level of the resources was 9 *±* 2.6. The average reading level for FOG and Flesch Reading Ease was Grade 10 difficulty, and the average reading level for FKGL and SMOG indices was Grade 7 difficulty. A reading grade two to three grades higher for the FOG and Flesch reading scale was consistent throughout the resources (Fig. 2). We observed no statistically significant association between resource characteristics (e.g., type of organisation, target audience) and readability scores (data not reported).

The two-page fact sheet entitled ‘Reducing the risk of workplace alcohol and other drug problems’, which was published by the Alcohol and Drug Foundation, obtained the highest elevated readability scores, measuring 11.6 for FKGL, 15.8 for the FOG index, 11.5 for the SMOG index, 14.5 for Flesch Reading Grade levels and an average readability of 13th grade level.

On the other hand, the two-page postcard entitled ‘What is FASD?’ obtained the lowest reading grade level measures, scoring at a 2nd grade ranking for FKGL with a rating of 1.7, 4th grade for the FOG index, a modest 3.5 on the SMOG index and 5th –grade reading ease for the Flesch Reading Grade level. Although this resource included 50% bulleted text, 50% visual to written text, and ‘adequate’ layout and typography, it did not contain any simple graphics or illustrations.

A two-page brochure called ‘Cracks in the ice: Information for staying safe’, developed by the Australian Government Department of Health and The University of Sydney Matilda Centre was a good example of a resource with easy readability and high scores for suitability and content. The average readability was 7th-grade level, with a FKGL score as low as 5th-grade level. Furthermore, this material addressed a range of AOD topics (55%), including the effects of using ice (methamphetamine), the impact of ice, drug interactions, tolerance and dependence, symptoms of overdose on ice, and contact information of the available support services. It also used ‘superior’ illustrations and infographics to convey valuable health information to the target population.

## Discussion

This study critically evaluated readily available online one to two-page AOD resources in NSW, Australia. We comprehensively evaluated the physical attributes, visual and written communication, thoroughness and content, and readability levels of the resources. We found that most resources demonstrated higher readability grade levels, lacked the use of graphics and infographics, and extensively used professional jargon. They displayed suboptimal typographic values, characterised by a need for more consistency in font size, font type and typographic cues. One recurrent drawback was the incorporation of complex sentences with embedded information, impeding comprehension. Despite these limitations, the resources adopted diverse formats such as brochures, factsheets, pamphlets, and postcards. While most resources targeted specific populations, some resources needed more content coverage specific to the target audience. Surprisingly, only three resources involved consumers in the resource development process. Nevertheless, positive attributes included purposeful headings, effective use of subheadings, commendable layout factors, the incorporation of colours, the use of active voice and evidence-based information related to AOD use. This highlights the need for organisations to co-design resources with consumers to improve suitability, content and readability.

This study stands out in its comprehensiveness compared to previous studies that focused on patient educational resources. For instance, El-Haddad et al. [22] examined the readability and content of lifestyle education resources for weight management in Australian general practice, while Hansberry et al. [26] assessed the readability of patient education materials in surgical subspecialties in America. However, both these studies overlooked the examination of textual characteristics such as the use of medical jargon, bulleted text, the percentage of visual to written text, layout, and typographic factors [26], limiting the resources to three or four messages, the complexity of sentences, and use of active and passive voice, all of which are crucial for patient comprehension. In contrast, the current study meticulously assesses all these criteria. While prior studies conducted by Amanda et al. [27] evaluated AOD resources targeted explicitly towards Aboriginal and Torres Strait Islander peoples, this study makes a significant advancement by being the first to evaluate all resources available for general AOD users, their families and caregivers in NSW, Australia.

The findings from the content analysis suggest the need for increased emphasis on specific topics, particularly alcohol and drug use during pregnancy and breastfeeding. Literature suggests that such information has the potential to bring about significant behaviour changes in families [48]. Furthermore, there was a noticeable absence of information regarding symptoms and harm minimisation during overdose and withdrawal. Existing research indicates that educating consumers on overdose management can effectively reduce the number of fatalities associated with such incidents [49]. Even though some resources considered a simplified descriptive approach suitable for the community, when some specific information is not included, there is a risk of either diminishing or exaggerating the dangers of substance abuse [17]. The absence of specific information creates ambiguity about the resource’s role in imparting knowledge and introducing behaviour change. This emphasises the importance of involving consumers in resource development and practice.

The results can also be interpreted through Nutbeam’s model of health literacy, which differentiates between functional, communicative (interactive), and critical health literacy [50]. Most AOD resources in our study primarily addressed functional health literacy, providing basic factual information about AOD harms and harm minimisation. However, the frequent use of complex sentences and the limited use of pictures may impede comprehension for individuals with low literacy skills, particularly among marginalised groups who are overrepresented in AOD harm statistics [51]. Communicative health literacy, which enables individuals to actively engage with healthcare providers and apply information in different contexts, or critical health literacy, which empowers them to critically appraise information and make informed decisions [50] were largely absent, reflecting a missed opportunity to empower communities to advocate for structural change. Furthermore, these gaps align with broader health literacy debates that stress the importance of moving beyond simply providing information, towards fostering higher-level literacy skills that address inequities in health outcomes [52]. This gap is particularly important for marginalised groups, who may require resources that not only inform but also build skills for negotiation, advocacy and self-management. Integrating communicative and critical literacy principles – potentially through participatory design and co-production – could transform resources from passive information tools into active enablers of empowerment and behaviour change [53].

Consumer involvement in the development of health education resources is increasingly recognised as essential for creating materials that are relevant, engaging, and accessible, particularly for populations with low health literacy. Participatory design methods such as co-design workshops, user-centred design, iterative testing, and advisory groups are well-established strategies to ensure resources meet users’ needs and preferences, thereby enhancing health literacy [54–56]. These approaches aim go beyond passive consultation, fostering collaborative partnerships that empower consumers to shape content, format and delivery. Research in oral health and nutrition demonstrates that involving target populations in refining language, imagery and delivery formats improves cultural appropriateness, relevance and user engagement, which leads to better comprehension and uptake [57]. Similar benefits have been observed in other health contexts; for example, a recent HPV health literacy program for youth employed co-design workshops and iterative prototype testing, resulting in clearer, culturally relevant materials and higher engagement [58]. Despite this evidence, our study found that only a small number of AOD resources (*n* = 3) incorporated consumer involvement. Participatory design principles are required throughout the development and review process to address this gap. Strategies include involving consumers early to identify priority topics and barriers, co-creating content to ensure plain language and cultural relevance, and using iterative prototyping with targeted feedback from end-users. Involving peer educators or individuals with lived AOD experience within development teams can further ensure authenticity and trustworthiness [55, 59]. Additionally, resources can also be tailored for specific communities, such as Aboriginal and Torres Strait Islander peoples, culturally and linguistically diverse groups, or youth, through tailored language, imagery and examples [54, 59]. Finally, involving consumers in setting evaluation criteria and interpreting results helps ensure resources are not only informative but also empowering, supporting functional, interactive and critical health literacy. This participatory approach would transform AOD resources from static information tools into responsive, user-centred interventions capable of driving sustained behaviour change. Collectively, these strategies underscore the importance of consumer involvement and offer practical guidance for improving AOD resource development.

Consumers, including those with good reading skills, generally prefer materials that are easy to read over challenging materials [37]. The simplification of materials involves two key aspects: design and writing [60]. The analysis of visual elements in this study, using SAM criteria, indicates significant room for improvement in the assessed resources. Despite established literature, including randomised control trials by Delp and Jones [61], emphasising the advantages of graphics, it is disconcerting that three-quarters of the assessed resources in our study lacked illustrations. An Australian study on Vietnamese-speaking mothers reported that leaflets with illustrations increased understanding and recall [16]. These findings emphasise the important role of incorporating visual elements in making health information accessible and understandable, particularly for individuals from culturally and linguistically diverse (CALD) backgrounds, who face language and literacy barriers [16, 62]. To address this, educators should not only acknowledge the potential of pictures to support key points but also consider minimising distracting details, using more simple drawings, and closely linking pictures to text or captions [60, 63]. Other critical considerations for future work in this area include involving individuals from the intended audience in designing pictures, and having health professionals, rather than artists, plan the visual elements [63].

Readability is one of many factors that determine the effectiveness of written information in improving a patient’s understanding of health-related issues. This study utilised four well-established readability indices, widely used in various healthcare domains, including surgery [26] and dentistry [23, 24], weight management [22] and now applied in the context of public health. The findings revealed that most of these resources surpassed the 6–8 grade readability recommended by NSW Health, averaging 9th-grade level. These findings resonate with the observations in existing literature [21, 24], indicating a considerable challenge for patients with low health literacy skills, particularly older individuals, those from CALD backgrounds and those with lower socioeconomic status, who make up 60% of the Australian population [15]. Moreover, the readability grades obtained for FOG and Flesch Reading Ease were two to three grades higher than the FKGL values. This is because FKGL values assess the readability at a 75% comprehension level [46]. It is crucial to note that the mere pursuit of a low reading grade level may not guarantee the efficacy of a resource. For instance, a two-page postcard entitled ‘What is FASD?’ achieved the lowest reading grade level measures. Despite its low readability grade levels, it covered only 11% of the AOD topics. While it discusses the long-term effect of alcohol use during pregnancy, it fails to provide further contact information for available support services or provide further guidance for families and caregivers of pregnant women affected with AOD misuse, indicating poor content coverage. Additionally, it lacked in layout, typographic factors, and use of illustrations. On the other hand, a brochure named ‘Cracks in the ice: Information for staying safe’ achieved low readability grade levels (averaging at 7th –grade level) and scored highly in suitability and content. This resource addressed a comprehensive range of AOD topics (55%), utilised ‘superior’ illustrations and infographics, and included valuable information on the effects of using drugs, drug interactions, symptoms of overdose and information on the available support services in NSW, highlighting that it is possible to develop AOD resources with low readability without compromising on the content.

Comparable challenges have been documented in the United States, where several studies have found AOD-related resources to be written well above recommended readability levels. For example, online patient materials for Alcohol Use Disorder averaged a 12th-grade reading level, with none meeting the NIH and AMA recommendation of 6th grade or below [64]. Similar results were observed in printed materials from substance use treatment programs, which averaged an 11.8-grade level [21]. These findings mirror our results, suggesting that the problem of overly complex AOD resources is not unique to Australia and reflects a broader, international gap in aligning resource design with health literacy principles.

### Strengths and limitations

This study surpasses previous research in Australia by comprehensively evaluating AOD resources, considering various domains such as readability and suitability of the visual and written elements, by examining the type and relevance of graphics used, use of jargon, writing style, active voice, bulleted text, content analysis, consumer involvement, target audience and physical characteristics of the resources.

This current study has a few limitations that need to be acknowledged. Firstly, the study primarily focused on online resources, neglecting hard-copy resources and web content. Nevertheless, the substantial sample size comprehensively represents AOD resources in NSW. Secondly, we evaluated resources specific to NSW, and the findings may not represent resources in other Australian regions. Thirdly, English-language resources were exclusively examined, potentially neglecting materials in local languages for individuals from CALD backgrounds. Another limitation of this study is the reliance on an automated web-based tool for assessing readability. Automated readability scores can be inconsistent due to variations in algorithms, text formatting, and linguistic nuances [65]. Finally, the study’s criteria selection may have overlooked factors influencing readers’ opinions.

### Recommendations and implications

The recommendations derived from our findings aim to guide AOD services in creating resources that are both informative and easy to understand for individuals with, or at risk of, AOD issues, as well as their families and carers. They offer valuable guidance for government and not-for-profit organisations developing materials for diverse audiences.

First, AOD resources should be readily available online to maximise reach and accessibility. Given that 80% of Australians use the internet for health information, online platforms provide a critical avenue for engagement across varied populations [66]. Second, limiting AOD resources to three or four key messages may improve usability, as consumers often prefer concise materials [15]. Third, standardised protocols should guide resource development, taking into account key quality dimensions, such as readability, suitability and content. Incorporating simple graphics alongside text can further enhance comprehension. Fourth, the absence of health literacy data on AOD users in Australia highlights the need for targeted research to ensure materials match the literacy skills of their intended audience. Fifth, policy makers and health agencies should embed participatory design processes, including the involvement of people with lived AOD experience, in the design, testing and evaluation of resources. This would help ensure materials are culturally relevant, accessible and responsive to the needs of marginalised communities. Finally, government and health agencies should adopt standards that require AOD resources to explicitly address all three levels of health literacy. While enhancing functional health literacy through clear, plain language is essential, strengthening communicative health literacy by adding interactive features, dialogue prompts and guidance on engaging with health professionals, and fostering critical health literacy by including skills for evaluating information, recognising misinformation and advocating for change, are equally important. Integrating these elements can transform AOD resources from static information tools to dynamic, user-centred interventions that empower individuals, promote self-advocacy and drive sustained behaviour change, particularly for marginalised populations.

## Conclusion

The research study reveals considerable variability in the physical attributes, visual and written communications, thoroughness and content, and readability grade levels of AOD resources available in NSW Australia.

The resources varied in format and included brochures, factsheets, pamphlets and postcards. Despite this variety, a noticeable gap was the limited content coverage specific to target audiences and the minimal involvement of consumers in the resource’s development process. This was accompanied by a lack of graphics and infographics, extensive use of professional jargon, and suboptimal typographic values. The frequent incorporation of complex sentences with embedded information and higher-grade readability levels, often surpassing the recommended 6–8 grade readability suggested by NSW Health, was identified as a recurrent drawback, potentially impending comprehension.

In conclusion, the existing AOD resources pose challenges in comprehension, particularly for individuals with lower health literacy. Given that AOD resources play a crucial role in supporting those with AOD issues or undergoing treatment, there is a pressing need to create evidence-based educational resources. This study emphasises the urgency of developing AOD resources that are easy to read and understand, specifically targeting individuals with lower literacy levels while maintaining high standards of content quality. Consumer involvement in the development of these resources plays a crucial role in addressing these challenges and improving the health literacy levels of AOD resources.

## Supplementary Information

Below is the link to the electronic supplementary material.

Supplementary Material 1

## Data Availability

Data sharing not applicable as no datasets generated and/or analysed for this study.

## Declarations

### Patient and public involvement

Patients and/or the public were not involved in the design, or conduct, or reporting, or dissemination plans of this research.
