# Supplementary material for: A comprehensive analysis of alcohol and other drug educational resources available in New South Wales, Australia for content, suitability and readability
Source: Addict Sci Clin Pract. 2025 Dec 2;20:93. doi: 10.1186/s13722-025-00615-5 (PMC12670872; doi:10.1186/s13722-025-00615-5)
Supplement: Supplementary file 1 — Supplementary Material 1 [file 13722_2025_615_MOESM1_ESM.docx]

# **Appendix 1. Inclusion and Exclusion criteria**

| **Inclusion Criteria** | **Exclusion Criteria** |
| --- | --- |
| 1. AOD materials which are written in the English language. | 1. Resources produced by Health Departments from other states and territories apart from NSW. |
| 1. Resources with a concise length of 1-2 pages. | 1. Resources that do not contain AOD-related messages regarding their use, management, and harm mitigation. |
| 1. AOD materials developed by government and not-for-profit organisations in NSW. | 1. Resources that only include information on accessing AOD services. |
| 1. AOD materials are readily available via the Internet. |  |
| 1. AOD materials are tailored for individuals who have or are susceptible to AOD disorders as well as their families and caregivers. |  |
| 1. Resources that are dedicated to addressing the adverse consequences of alcohol and illicit drug misuse, as well as strategies for managing and reducing harm. |  |

**Appendix 2. Equations for the four-readability metrics**

| **Indices** | **Formula** |
| --- | --- |
| Flesch Reading Ease | 206.835-(1.015×WL) -84.6 SL |
| Flesch -Kincaid grade level | (0.39×WL) +(11.8×)– 5.59 |
| Simplified Measure of Gobbledygook | 3+√PSW count |
| Gunning Fog Index | 0.4 (ASL+ percentage of PSW) |
| WL - words/sentence length  SL - syllables /words  ASL - average sentence length  ASW - average number of syllables per word  PSW - polysyllable words | |

**Appendix 3. Interpretation Table for Flesch Reading Ease Scores**

| **Description of writing style** | **The score for reading ease** | **Estimated reading level** |
| --- | --- | --- |
| Very Easy | 90 to 100 | Fifth Grade |
| Easy | 80 to 90 | Sixth Grade |
| Fairly Easy | 70 to 80 | Seventh Grade |
| Standard | 60 to 70 | Eighth and Ninth Grades |
| Fairly Difficult | 50 to 60 | Tenth to Twelfth Grades |
| Difficult | 30 to 50 | Thirteenth to Sixteenth (College grade) |
| Very Difficult | 0 to 30 | Above sixteenth (College Graduate) |
